# Molecular Research in Urology 2014: Update on PET/MR Imaging of the Prostate

**DOI:** 10.3390/ijms150813401

**Published:** 2014-07-31

**Authors:** Axel Wetter

**Keywords:** PET/MRI, prostate, multi-parametric MR imaging

## Abstract

This article gives an overview of recent publications and potential indications of Positron emission tomography/ Magnetic resonance (PET/MR) imaging of prostate cancer.

During the past three decades, magnetic resonance (MR) imaging of the prostate has been a subject of profound radiological research with continuous technical innovations [1,2,3,4,5]. Nowadays, state-of-the-art MR imaging of the prostate implies a multi-parametric approach, in terms of combining different anatomical and functional MR sequences in order to get a deep insight into the underlying pathophysiological processes of prostate cancer [6]. Prostate cancers typically display a low signal in T2-weighted images, which makes them distinguishable from the normal and usually hyper-intense peripheral zone. However, also other entities like chronic prostatitis can show a hypo-intense signal in T2-weighted images. In addition, tumors that arise from the transitional zone are often difficult to detect, as the signal of the transitional zone is heterogeneous and may show changes caused by benign prostatic hyperplasia with mixed hypo- and hyper-intense signal alterations [7]. Therefore, a multi-parametric imaging approach seems to be very useful in order to get as much information as possible beyond pure anatomical information. The three main components of multi-parametric MR imaging in prostate cancer imaging beside T2-weighted imaging are: diffusion-weighted imaging (DWI), dynamic contrast-enhanced imaging (DCE) and proton-MR-spectroscopic imaging (^1^H MRSI). Several original research articles showed that a combination of these functional imaging modalities can improve diagnostic accuracy of MR imaging [8,9]. Therefore, modern MR imaging protocols for visualization of prostate cancer should comprise these functional MR imaging sequences. To achieve a standardization of MR imaging of prostate cancer, the European Society of Urogenital Radiology (ESUR) published guidelines of how to perform a sufficient MR examination consisting of a multi-parametric approach [10]. The ESUR guidelines furthermore proposed a structured reporting scheme, called PI-RADS, where imaging findings from anatomical and functional MR sequences are weighted with respect to their likelihood of indicating malignancy.

PET and PET/CT imaging of prostate cancer with the radiotracers [^11^C] or [^18^F] choline has been examined to a large extent during the past years; however, with partly controversial results. A large meta-analysis defined possible indications of PET/CT examinations of the prostate including initial staging of patients with newly diagnosed prostate cancer and high-risk of nodal extension and suspected recurrent prostate cancer after surgery or radiotherapy indicated by a rising PSA-level. Another indication of a PET/CT scan of the prostate might be a history of negative biopsies in patients with a strong suspicion of prostate cancer to plan a focused re-biopsy in carefully selected cases [11]. However, a major disadvantage of prostate imaging with PET/CT is the limited soft tissue contrast provided by the CT scan, thereby impairing lesion targeting.

As PET imaging and multi-parametric MR imaging of the prostate represent imaging modalities based on different physical, technical and biological principles, an additional benefit might be expected if both modalities are combined. Since the introduction of commercially available integrated PET/MR scanners that are capable of simultaneous acquisition of the PET and MR signal, some research has been performed to elucidate the potential of this new technology. Initial results proved the feasibility of simultaneous [^18^F] choline PET/MR imaging in patients with prostate cancer [12]. The integration of a PET and MR device into one system is technically challenging, as both might interfere. Therefore, studies that examine both components are needed. For both [^11^C] and [^18^F] choline was shown that PET and MR image quality was sufficient and without significant differences in the detection of hyper-metabolic lesions and with excellent correlation of standardized uptake values derived from PET/MRI and PET/CT [13,14]. In comparison to PET/CT, PET/MRI was shown to perform better in lesion allocation [14]. As the technical feasibility has been proven, added value of integrated PET/MR in prostate cancer imaging needs to be identified. A recently published prospective trial revealed a clear superiority of diagnostic accuracy of combined PET/MRI over MRI or PET alone in primary prostate cancer [15]. Although this study did not work with real simultaneous acquisition of PET and MRI but with post-hoc fusion of sequentially acquired PET and MRI datasets, this result might be ground-breaking regarding the use of integrated PET/MRI in primary prostate cancer. This study is, however, limited by the fact that a multi-parametric MR approach was not applied. Further promising results of the combined use of [^18^F] choline PET/MRI were reported for detection of prostate cancer relapse after external beam radiotherapy [16]. The combination of functional MR sequences like DWI and DCE imaging with the metabolic information of PET offers great potential, as it allows a detailed quantitative characterization of tumor lesions with measurements of standardized uptake values (SUVs), apparent diffusion coefficient (ADC) values and enhancement curves during one examination (see Figure 1 and Figure 2). In this context, first results have been published for quantitative imaging of primary prostate cancers [17] and for bone metastases from prostate cancer [18]. A novel development is a new tracer for prostate cancer imaging, a [^68^Ga] labeled prostate-specific membrane antigen (PSMA) ligand. Recent studies demonstrated the technical feasibility of PET/MR imaging with this new tracer and found a superiority of PET/MRI over PET/CT by using this tracer [19,20]. However, MR based scatter correction with the [^68^Ga] labeled tracer yielded some difficulties and requires further investigation before routine use [20].

Figure legends: Images from a patient with biochemical failure after radical prostatectomy (Figure 1) and from a patient with histological proven prostate carcinoma (Figure 2). [^18^F] choline PET/MR imaging was performed after PET/CT. The images illustrate the combined findings of anatomical sequences together with diffusion-weighted imaging and PET imaging.

**Figure 1:**
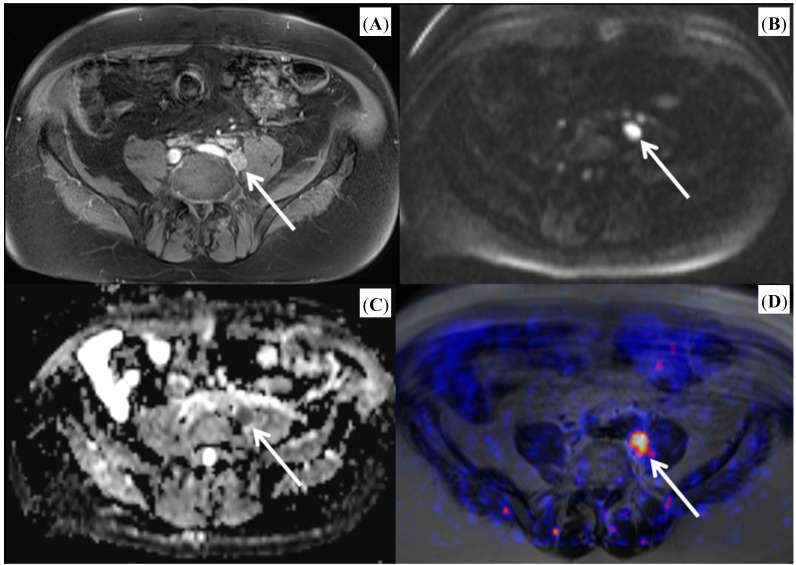
Patient with a lymph node metastasis from prostate cancer. (**A**) T1-weighted image after administration of contrast medium showing an enlarged left iliac lymph node with contrast enhancement; (**B**) Diffusion-weighted images revealing a diffusion restriction of the lymph node indicated by a hyper-intense signal; (**C**) Corresponding apparent diffusion coefficient (ADC) map with hypo-intense depiction of the lymph node; (**D**) Fused PET/MR image with pronounced choline uptake of the lymph node. (Arrows indicate lymph node metastasis).

**Figure 2:**
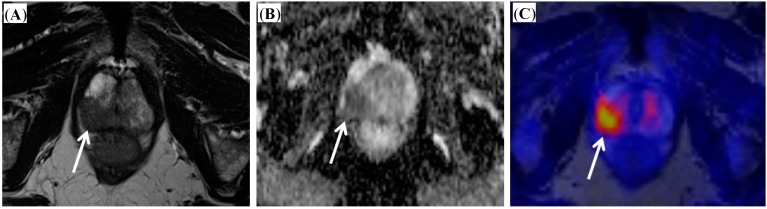
Patient with biopsy proven prostate carcinoma (Gleason score 4 + 3 = 7). (**A**) T2-weighted image presenting a hypo-intense lesion of the right peripheral zone; (**B**) ADC map indicating restricted diffusion within the tumor; (**C**) Fused PET/MR image depicting pathologically increased choline uptake of the tumor. (Arrows indicate prostate carcinoma).

In conclusion, the results published so far on PET/MRI of prostate cancer are promising and justify further research in this field. It will be necessary to plan and conduct large prospective studies in order to ascertain the additive value of PET/MRI in comparison to the available diagnostic tools in prostate cancer diagnostics.
